# Supplementary figures and images for: A neural network model enables worm tracking in challenging conditions and increases signal-to-noise ratio in phenotypic screens
Source: PLoS Comput Biol. 2025 Aug 8;21(8):e1013345. doi: 10.1371/journal.pcbi.1013345 (PMC12360645; doi:10.1371/journal.pcbi.1013345)

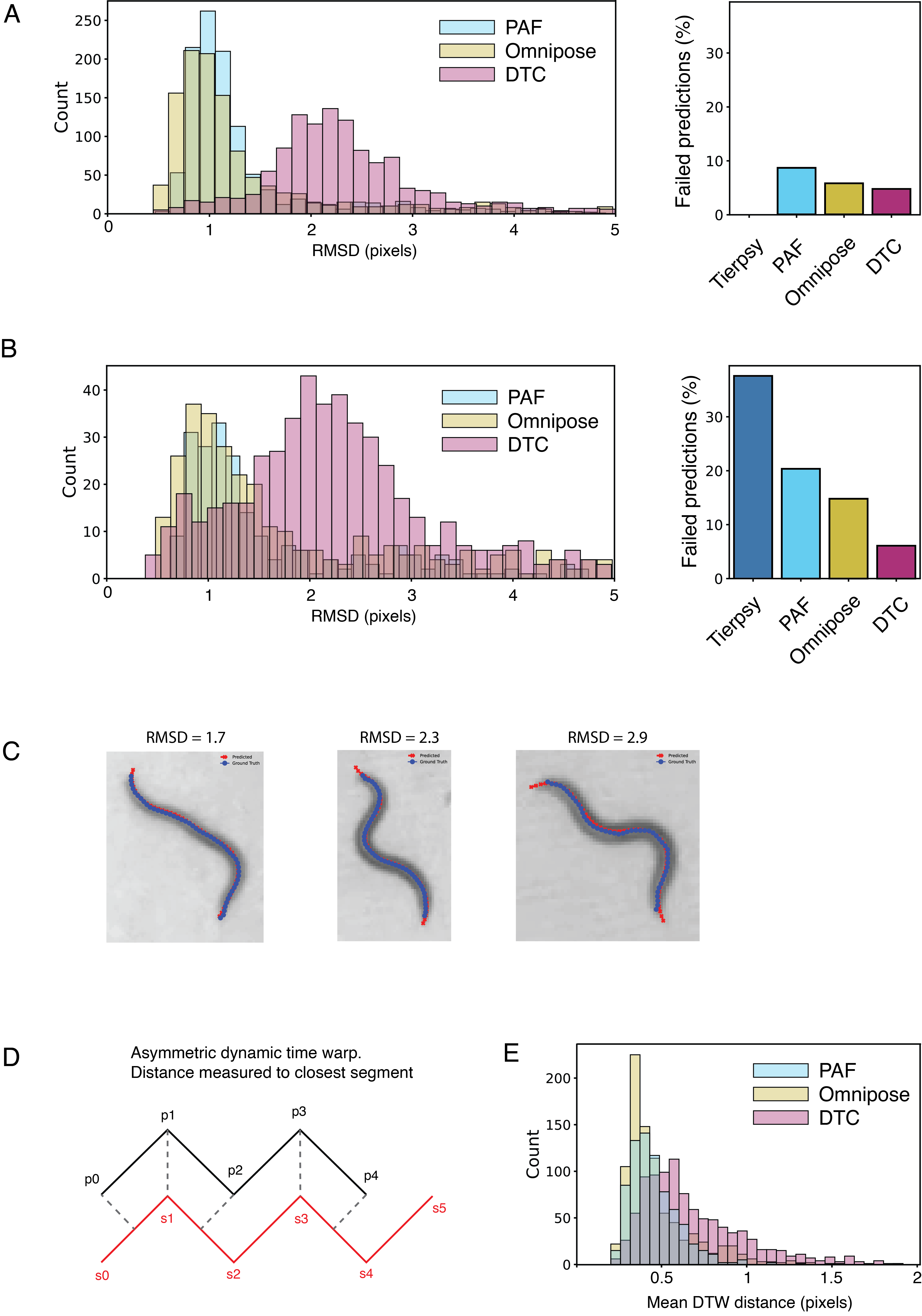

Supplement: S1 Fig — (A) Root mean square deviation between model predictions and test data for ‘easy’ cases which were skeletonized by Tierpsy. By construction the number of failed predictions for Tierpsy is zero. (B) The same as A but using manually annotated skeletons as the test data. These data have more difficult cases, but there are still a substantial number that Tierpsy is able to make predictions for (Tierpsy fails around 35% of the time on these data). (C) Examples showing manual annotations (blue) along with DTC predictions in red with different values of RMSD. In all cases, the predicted skeletons are close to the worm midline and the RMSD is driven mostly by overshooting at the head and/or tail. (D) Instead of measuring the point-to-point RMSD, we can measure the distance to the nearest portion of the test segment for each of the model predictions. (E) The mean distance computed as shown in D is more similar across the models although DTC still performs slightly worse. (PNG) [file pcbi.1013345.s001.png]

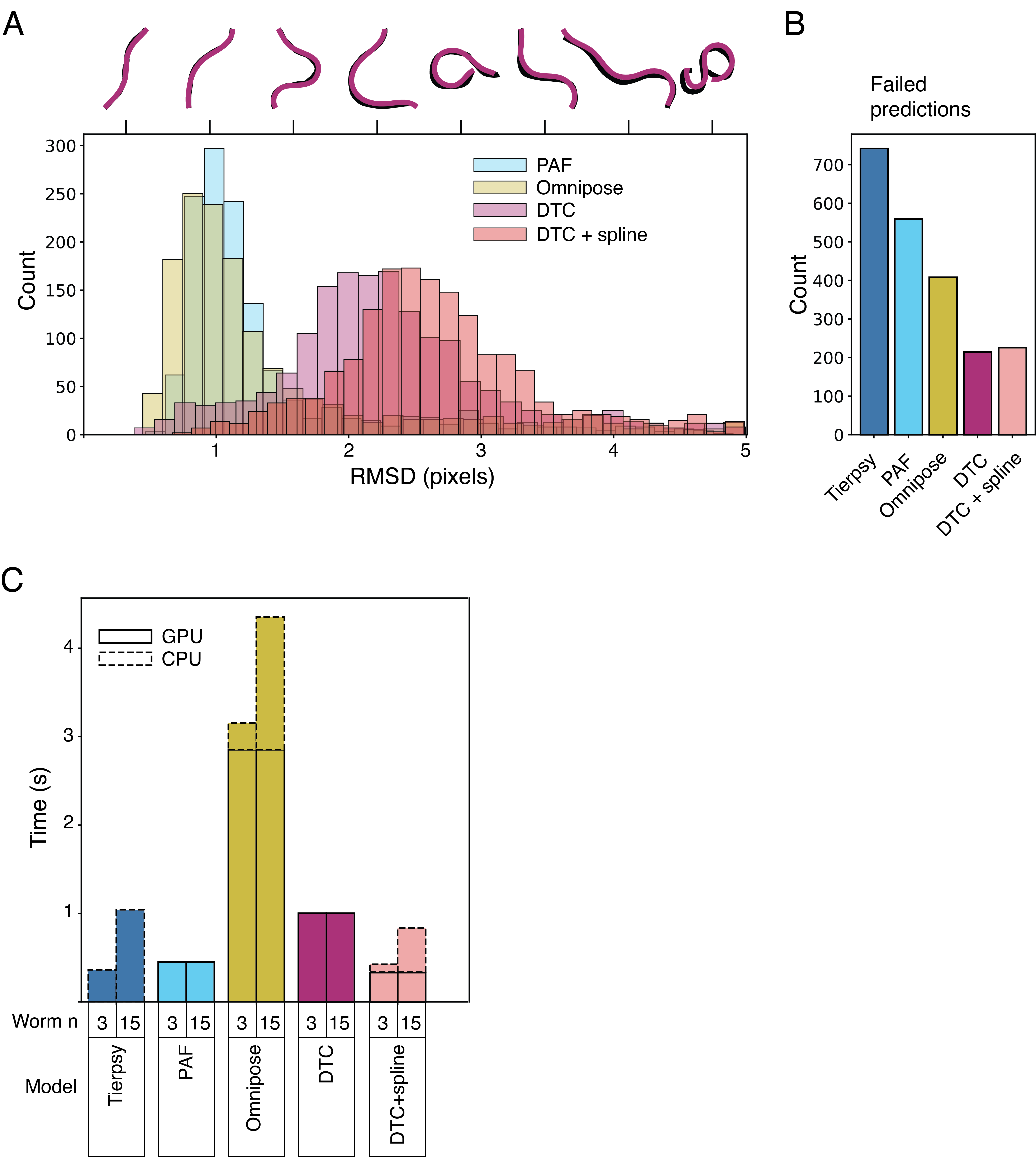

Supplement: S2 Fig — (A) Histograms of root mean square deviation between held-out skeletons and predicted skeletons for each tested model, here including ‘DTC + spline’ in which only every third frame is tracked using DTC and skeletons for intermediate frames are interpolated using a 3D smoothing spline. The vertical dashed line shows the RMSD between three manual annotators. Skeletons above the histogram are examples that illustrate the corresponding RMSD visually. (B) Bar chart showing the number of cases in the held-out test data where a model fails to make a prediction (e.g., Tierpsy fails on coiled worms or a neural network model does not identify any worms above a confidence threshold). (C) Computation time per input frame for the different models as a function of worm number. Tierpsy only uses CPU computation while Omnipose uses GPU and CPU because we use Tierpsy’s skeletonization algorithm to convert segmented regions to skeletons. DTC + spline uses both GPUs for predicting skeletons on the subsampled frames and CPUs for fitting smoothing splines to interpolate the missing frames. The advantage is modest for videos with 15 worms/well (which is nominally 240 worms/video since each camera records 16 wells on a 96 well plate). (PNG) [file pcbi.1013345.s002.png]

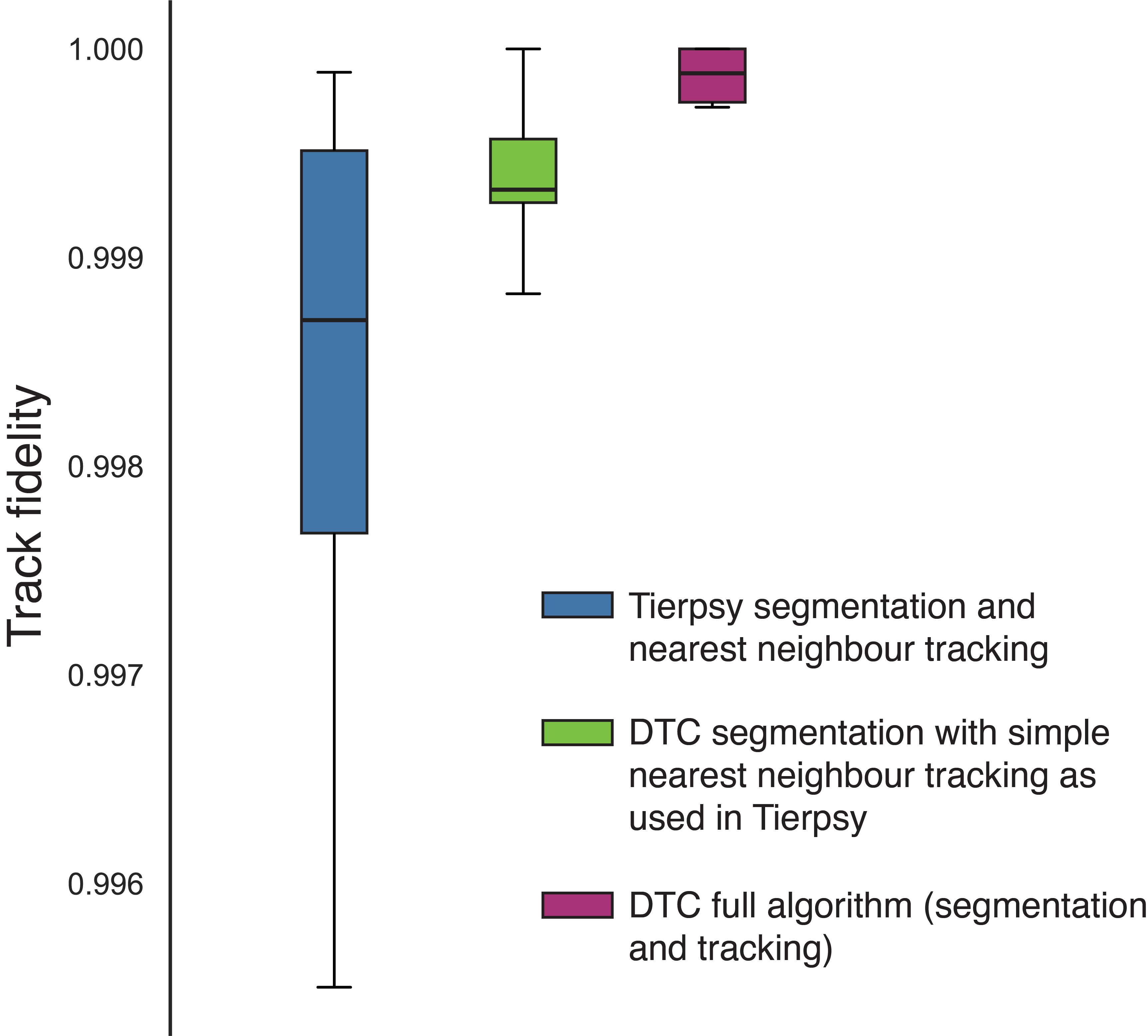

Supplement: S3 Fig — (PNG) [file pcbi.1013345.s003.png]
