# Supplementary material for: A neural network model enables worm tracking in challenging conditions and increases signal-to-noise ratio in phenotypic screens
Source: PLoS Comput Biol. 2025 Aug 8;21(8):e1013345. doi: 10.1371/journal.pcbi.1013345 (PMC12360645; doi:10.1371/journal.pcbi.1013345)
Supplement: Attachment — Submitted filename: reviewer-response.pdf [file pcbi.1013345.s008.pdf]

Reviewer comments are in blue; our responses are in black.

We thank the reviewers for taking the time to assess our work and for providing helpful comments. We have updated the manuscript and analysis to address their concerns as detailed below. Text added to the manuscript is highlighted.

Reviewer #1: The authors present significant progress in enabling the tracking of crawling nematodes in challenging environments. In my view, the results are novel and represent the current state-of-the-art in tracking. However, the presentation of the paper could be improved. I recommend that the authors carefully revise the manuscript to enhance its professionalism and clarity. Beyond that, I have specific comments that I hope the authors will consider:

- In order to understand RMSD, it is important to be able to understand your data. How much of this data was hand-annotated? If I understand your definition of "Inter-annotator RMSD" correctly, if all the data was hand-annotated, then all the ML models would have RMSD with mean around this value (~4.5 px), since the labels themselves would have this variance. Some details on the data and a discussion of this is warranted.

Thanks for catching this. The short answer is that we made a mistake when comparing the data annotated by multiple users. We forgot the interpolation step to make 49 equally spaced points which artificially inflated the RMSD for this comparison only. This is now fixed in the main figure and the median RMSD for the inter-annotator error makes more sense.

As you guessed, most of the test data (similar to the training data) comes from ‘easy’ cases that were skeletonized by Tierpsy. The reason we didn’t initially catch our mistake is that we thought the models were better at reproducing the Tierpsy annotations than humans (which is plausible) and this skewed the data. However, when we split the data (new Supplementary Figure 1), it was clear that the RMSD on the hand annotated cases still looked better than the inter-annotator variability.

We have now included some examples in Supplementary Figure 1 showing hand-annotated and model-predicted skeletons showing that DTC often misses the end points of the worm but otherwise traces the centreline fairly accurately. This is borne out by a different distance measure that’s now also included in Supplementary Figure 1 and discussed briefly in the main text.

- The difference in accuracy between omnipose and DTC is interesting. I suppose this is due to omnipose working directly on pixels, but DTC predicting coordinates. Have you tried any type of post-processing refinement? For instance, the DTC output could initialize an "Active Contour Model" or similar that could adapt to the pixels. Could be discussed.

We haven't tried this but it's a good idea. Interestingly, after submission a preprint was published (https://doi.org/10.48550/arXiv.2503.14525) which we now cite in the discussion that shows that DTC output can be improved using differential rendering (which the authors show works better than an active contour on these data).

- You write "Because the worms do not move, DTC is unable to take advantage of temporal information to resolve the coiled shape.". Earlier you mentioned that you take the 11-frame window to mean different durations during training. Could you not do a similar trick during inference? i.e. somehow make the windows longer when there is less motion? Could be discussed at least.

We agree and indeed considered taking motion into account during inference. We have added this to the discussion and will think about including it in future work.

- You mention original DT was trained with synthetic data. Was is the state-of-the-art of crawling worm simulations?

One of the cited methods, WormPose, uses semi-synthetic training data. For crawling motion, the state of the art is probably either our work on using LSTMs to generate trajectories or the more recent work from Costa et al. (2024). However, neither method is trained on overlapping worms and some of the hardest cases where worms stay closely aligned while doing small motions forwards and backwards aren't well captured. Furthermore, even given perfect trajectories, there is still the question of generating the corresponding images. We have included some discussion on this point at the end of the paper.

- Finally, maybe I misunderstood something, but I think the title, "An improved neural network model enables worm tracking in challenging conditions and increases signal-to-noise ratio in phenotypic screens", misrepresents the paper. As far as I can tell, the authors used a preexisting neural network, and did not make changes to this, but rather used a new type of data. If that is indeed the case, I would instead suggest "A neural network model enables worm tracking in challenging conditions and increases signal-to-noise ratio in phenotypic screens" for the title.

You're correct that we used the previously published architecture. We believe the most common usage for 'model' in this context includes both the architecture and the weights and so the network trained on new data is an improved model. Nonetheless, we have updated the title as suggested.

Minor:

Fig. 1A: The arrows confused me slightly. Could <------> be replaced with |------|? (if you agree)

Thanks, we've updated the figure.

Reviewer #2: The authors introduce DeepTangleCrawl method to infer the skeletons of overlapping C. elegans in images. The method is based on previously published DeepTangle approach that was developed on images of swimming worms. Here it was retrained on images of crawling C. elegans whose poses are more challenging to resolve. The paper is well written and clear I have however a few concerns about the types of comparisons the authors do to assess the method's performance.

1. Both Omnipose and PAF were not developed specifically for C. elegans. Additionally, a keypoint-based method such as PAF is not well suited for resolving the pose of an organism without skeleton and limbs. I suggest that a C. elegans-specific segmentation and pose estimation method (some are cited in line 45) would be a more fair comparison of the performance.

Omnipose was developed in part for C. elegans, at least in the sense that the original publication includes both worms and bacteria as use cases. Still, we agree that there are more models to consider, including other worm-specific models. When we started the project, these were not yet available and we had already implemented several models by the time they were available. We made an exception for DeepTangle because of its impressive results on swimming worms.

While we agree that these worm-specific models would be useful for comparison, we don't have the resources to implement more benchmarks for this manuscript (the first author has moved to a new position).

In our defence, the level of benchmarking we show here is more extensive than is typical in the field. For comparison, the three worm-specific methods we cite (Deep-worm-tracker, WormSwin, and DeepTangle) have no external benchmarks at all and only quantify the effect of internal model choices.

2. line 52: "achieving a new state-of-the-art on challenging worm tracking data compared to two instance segmentation approaches." Segmentation and tracking are 2 different tasks and why would one compare segmentation methods in tracking performance? It is again not a fair comparison as tracking methods are optimized for tracking and segmentation methods for segmentation. Also, which instance segmentation methods the authors refer to?

We agree this is confusing and have made the comparisons clearer in the introduction. In the first part of the paper, we are only comparing the segmentation performance of the models. When we found the segmentation performance was good for DTC (segmented the highest number of challenging cases with acceptable accuracy), we only compared tracking results between DTC and Tierpsy.

3. I am also curious to know whether the method allows to segment tightly coiled worms, such as on Fig. 2c if they are not coiled persistently? Do the authors have an example where motion of the worm allows to disentangle the tightly coiled posture?

DTC still fails for the tightest coils even if there is a nearby frame without coiling. We have updated the Figure 2 caption to say "DTC fails when worms are fully coiled in circles or in tight parallel contact for an extended time" and the results to say "When these slow-moving coilers do straighten within a clip available to DTC, it is often able to estimate the pose in nearby coiling frames although it still fails for the tightest circular coils." We suspect that with more training data on the highly coiled worms it could work in principle, but these are not very well represented even the in the hand-annotated data.

4. How is the tracking performed? There is no information on how the individual poses are linked into trajectories. Is it the same strategy as Tierpsy? In that case the tracking performance difference is due uniquely to the improved pose estimation.

We used the same tracking algorithm as in the DeepTangle paper. We have now included an additional plot (Supplementary Figure S3) showing that both the detection and tracking algorithm contribute. We used the detections from DTC and fed them into a nearest neighbour tracker. This shows intermediate performance compared to the full DTC algorithm and the legacy Tierpsy algorithm.

5. Is there tracking groundtruth for these data? Can the authors present any relevant tracking metrics such as percentage of correctly tracked or the MOT metrics? Otherwise, I question whether the authors can indeed make a statement about the method's tracking performance. There is simply more, better-resolved poses which allow to generate a higher number of longer trajectories, which are not necessarily correct.

This is an important point. We had inspected many trajectories but not quantified the performance. We manually corrected the tracks in several videos and used this as ground truth for evaluating both trackers. Performance was quantified using the ratio of mismatches (the fraction of times tracks were incorrectly switched compared to the total number of ground truth objects in all frames). This shows that the longer trajectories produced by DTC are also more accurate and that the improvement is larger for higher worm densities. The plot is the new Fig. 3F.

6. There is a typo "identify" in caption of Fig. 3

Thanks, this is fixed.

Reviewer #3: The submitted article reports the development of an improved deep learning based worm tracker that delivers better signal to noise ratio and also carries segmentation and tracking on agar surfaces and thick lawns. The work, called DeepTangleCrawl (DTC), takes off from the earlier DeepTangle developed by A. Alonso and J. B. Kirkegaard Commun. Biol (2023). The manuscript is well written and presented. However, I have the following specific comments.

Specific comments:

1. On line 85: Specifically mention which challenging cases were annotated manually.

We have updated the text to mention the categories of challenging cases that were annotated manually. Please let us know if we've misunderstood this comment. We can't include the full set of cases in the text, but these are in the dataset published on Zenodo.

2. The work uses 11-frame clips for training purposes. Is this optimal? A comparison or sense of how many frames should be enough needs to be mentioned.

We don't know if it's optimal. There's a trade-off between how much context the model is given and computational time. This is mentioned in the original DeepTangle paper and we didn't evaluate the choice in extensively. We tried a few values in small early tests and didn't see results that made us question the original authors' choice of 11.

3. Line 88-89 mentions "Compared to the original DeepTangle model, we increased the dimension of the late space representing worm shapes from 12 to 72." What is late space? And, it is not clear what 12 to 72 means. What units are these in?

Apologies, that should have been the number of principal components representing the worm shapes (now fixed in the text). It's the number of dimensions of the space that we are increasing from 12 to 72, so those numbers themselves don't have associated units.

4. As per Fig. 2A in section "Pose estimation accuracy", RMSD is high for the DTC. Since RMSD computes the deviation in the minimum distances between the predicted and labelled splines. Intuitively, a better algorithm should reduce RMSD. What does an increased RMSD then mean?

It's correct that the RMSD is higher for DTC than for the other methods which does mean it predicts the skeleton coordinates less accurately. However, the performance is still good (the example skeletons and corresponding errors show that the worm shape is well-approximated when the RMSD is, for example, 3 pixels). The important advantage for DTC is that it is able to make predictions for more difficult cases than the other models (Figure 2B). In other words, when the other models make a prediction, they are closer to the worm midline than DTC, but there are more cases where the other models fail completely. The new Supplementary Figure 1 shows more examples with different values of the RMSD to help build intuition for what a given value means.

5. DTC uses the same code as DeepTangle. It is not clear what additions or improvements are made to the original DeepTangle that lead to improved segmentation in crawling worms? This should be made explicit and highlighted appropriately.

The main change is the training data, but we also changed some hyperparameters. We have updated the introduction to read "Here we train DeepTangle on a new dataset including the more complex postures and long overlaps of crawling worms and show that the resulting model, DeepTangleCrawl (DTC), can track coiling and overlapping

worms, achieving a new state-of-the-art on challenging worm tracking data compared to two instance segmentation approaches."

6. Line 126 mentions that all models fail in cases of complex overlap. What could be a potential solution?

We don't really know. There will always be cases that are complex enough (a compact aggregate with dozens of worms, for example) that skeletonisation from brightfield data won't work. To push to more complex cases, our first choice would be more training data since we don't think we've saturated the current architecture. Manual data is difficult for complex cases and so cracking the synthetic data challenge is probably the most promising near-term approach.

We now mention this in the discussion.

7. Video Movie_S1.mp4 shows the segmentation in a video, but also shows that there are frame drops. How often are the frame drops, which may lead to ID change or switch in worm ID? This, therefore, needs to be quantified and statistics should be reported.

The number of failed predictions on the test data is shown in Figure 2B. We have converted this to a percentage of failures which is more interpretable. We have also now included a tracking ground truth data set and the frequency of ID changes (Figure 3F).

8. Finally, a general comment I have is that the main achievement or improvement of DTC is better segmentation of worms in challenging poses, such as self overlapping, multiworm overlaps, coiling, etc. However, the term "pose estimation" used in the manuscript seems to be misleading, as on first reading, the impression was that it would be able to distinguish between poses such as omega turns, reversals, etc. However, the main point here is that it is merely able to carry out the detection of the worm even when they are in these poses. It would be better to state or highlight this clearly.

The common usage of pose estimation is about position and orientation (and joint angles for humans and animals) rather than behaviours like omega turns or reversals. We now define it when it first appears in the introduction.
